# The Role of Oxidoreductases in Determining the Function of the Neisserial Lipid A Phosphoethanolamine Transferase Required for Resistance to Polymyxin

**DOI:** 10.1371/journal.pone.0106513

**Published:** 2014-09-12

**Authors:** Susannah Piek, Zhirui Wang, Jhuma Ganguly, Adam M. Lakey, Stephanie N. Bartley, Shakeel Mowlaboccus, Anandhi Anandan, Keith A. Stubbs, Martin J. Scanlon, Alice Vrielink, Parastoo Azadi, Russell W. Carlson, Charlene M. Kahler

**Affiliations:** 1 School of Pathology and Laboratory Medicine, and The Marshall Center for Infectious Diseases, Research and Training, University of Western Australia, Perth, Western Australia, Australia; 2 Complex Carbohydrate Research Center, University of Georgia, Athens, Georgia, United States of America; 3 School of Chemistry and Biochemistry, University of Western Australia, Perth, Western Australia, Australia; 4 Monash Institute of Pharmaceutical Sciences, Monash University, Melbourne, Victoria, Australia; 5 ARC Centre of Excellence for Coherent X-ray Science, Monash University, Melbourne, Victoria, Australia; Public Health England, United Kingdom

## Abstract

The decoration of the lipid A headgroups of the lipooligosaccharide (LOS) by the LOS phosphoethanolamine (PEA) transferase (LptA) in *Neisseria* spp. is central for resistance to polymyxin. The structure of the globular domain of LptA shows that the protein has five disulphide bonds, indicating that it is a potential substrate of the protein oxidation pathway in the bacterial periplasm. When neisserial LptA was expressed in *Escherichia coli* in the presence of the oxidoreductase, *Ec*DsbA, polymyxin resistance increased 30-fold. LptA decorated one position of the *E. coli* lipid A headgroups with PEA. In the absence of the *Ec*DsbA, LptA was degraded in *E. coli*. *Neisseria* spp. express three oxidoreductases, DsbA1, DsbA2 and DsbA3, each of which appear to donate disulphide bonds to different targets. Inactivation of each oxidoreductase in *N. meningitidis* enhanced sensitivity to polymyxin with combinatorial mutants displaying an additive increase in sensitivity to polymyxin, indicating that the oxidoreductases were required for multiple pathways leading to polymyxin resistance. Correlates were sought between polymyxin sensitivity, LptA stability or activity and the presence of each of the neisserial oxidoreductases. Only meningococcal mutants lacking DsbA3 had a measurable decrease in the amount of PEA decoration on lipid A headgroups implying that LptA stability was supported by the presence of DsbA3 but did not require DsbA1/2 even though these oxidoreductases could oxidise the protein. This is the first indication that DsbA3 acts as an oxidoreductase *in vivo* and that multiple oxidoreductases may be involved in oxidising the one target in *N. meningitidis*. In conclusion, LptA is stabilised by disulphide bonds within the protein. This effect was more pronounced when neisserial LptA was expressed in *E. coli* than in *N. meningitidis* and may reflect that other factors in the neisserial periplasm have a role in LptA stability.

## Introduction

The obligate human pathogen, *N. meningitidis*, is the causative agent of transmissible sepsis and epidemic meningitis [1]. Although penicillin and cephalosporins are recommended for treatment, incremental sporadic increases in resistances to penicillin [2], rifampycin [3] and fluoroquinilones [4] have been detected and can cause periodic treatment failure. *N. meningitidis* is genetically conserved with the obligate human pathogen *N. gonorrhoeae* which is the causative agent of sexually transmitted gonorrhoea. Unlike *N. meningitidis*, *N. gonorrhoeae* has developed multi-drug resistance (MDR) phenotypes [5] which result in antibiotic treatment failures [6]. Since the rate of gonococcal disease in developing nations is approximately 10 times that of developed nations with approximately 106 million cases reported per annum, the world-wide spread of antibiotic resistant gonococci is of increasing concern [7].

One class of drugs which is being increasingly used for the treatment of MDR-Gram negative bacteria (GNB) causing sepsis is the class of cationic antimicrobial peptides (CAMPs), which includes the two clinically relevant compounds, polymyxin B and colistin (polymyxin E) and natural defensins such as LL-37 and protegrins. These compounds are generally considered to cause killing by lysis or by exerting a lethal oxidative stress response [8]. CAMPs have a cationic charge which enables them to bind to the negatively charged phosphate headgroups of the lipid A of LPS and accumulate in the outer membrane [9]. The electrostatic interaction stabilises the complex, enabling the polar face of the CAMP to align with the polar head groups of the lipid A and the lipophilic tail of the CAMP to insert into the membrane. The CAMP migrates across the periplasm and integrates into the inner membrane to form a pore, disrupting the phospholipid bilayer which results in cell lysis [9]. CAMPs consist of different structural classes which influences antimicrobial resistance profiles [10]. Although both *N. gonorrhoeae* and *N. meningitidis* are sensitive to protegrin and LL-37 [11], [12], they are both intrinsically resistant to polymyxin [13], [14].

GNB that are resistant to polymyxin substitute the negatively charged lipid A phosphate headgroups with positively charged moieties such as 4-amino-4-deoxy-L-arabinose (Ara4N). These residues disrupt the electrostatic interactions of the lipid A headgroups with polymyxin, reducing accumulation and integration of the CAMP in the outer membrane [9]. However, the intrinsically polymyxin resistant *Neisseria* spp. do not possess the ability to synthesise Ara4N [15]. Gonococci and meningococci produce similar lipopolysaccharide (LOS) structures which have a conserved inner core region composed of heptose and 3-*deoxy*-D-*manno*-2-octulosonic acid (KDO) attached to a lipid A moiety embedded in the outer-membrane [16]. A variable length α-chain that extends from the 4′ position of heptose I residue and is often composed of lacto-*N*-neotetraose forms the outer core region of neisserial LOS. In both species, the lipid A headgroups are decorated with phosphoethanolamine (PEA) by the lipid A PEA transferase, LptA [16], [17]. Both the 1 and 4′ headgroups of lipid A can be substituted with PEA and both residues are lost upon insertional inactivation of *lptA* [17]. Not only does the loss of PEA residues from lipid A result in the loss of resistance to polymyxin [13], it also results in sensitivity to complement mediated lysis [18] and a loss of the ability to attach and invade host cells [19]. The inactivation of LptA in *N. gonorrhoeae* results in the abrogation of colonisation in mouse and human models of infection [20]. In addition, LptA can be considered an important virulence factor in neisserial pathogens as it is absent in most commensal species and this is associated with reduced inflammatory potential of the lipid A from these species [21].

LptA is a member of the phosphoethanolamine (PEA) transferase family, YhjW/YjdB/YijP, and the structure of the globular domain has been recently solved by Wanty et al. [22]. LptA was shown to possess five disulphide bonds suggesting that it could be a substrate for the protein oxidation pathway [23]. *Neisseria* spp. contain up to three specialised oxidoreductases, DsbA1, DsbA2 and DsbA3 [23] that introduce disulphide bonds into proteins in the periplasm. DsbA1/2 have overlapping specificities and are required for the introduction of disulphide bonds into the type IV pilin proteins, PilE and PilQ [24]–[26]. However, DsbA3 has no known substrate. When oxidoreductases are inactivated in GNB, the loss of disulphide bonds in the substrate proteins results in decreased stability of the protein in the periplasm due to sensitivity of the proteins to periplasmic proteases [27]. To further understand the mechanisms governing LptA expression in *Neisseria* spp., we examined whether LptA stability is dependent upon the periplasmic protein oxidation pathway and whether this affected polymyxin resistance.

## Methods and Materials

### Bacterial strains and growth conditions

*E. coli* strains were grown either in Luria-Bertani (LB) broth or LB agar at 37°C. Meningococcal strains were grown with 5% CO_2_ at 37°C unless specified otherwise. Gonococcal base agar (GCA, Oxoid) was supplemented with 0.4% glucose, 0.01% glutamine, 0.2 mg of co-carboxylase per litre, and 5 mg of Fe(NO_3_)_3_ per litre. GC broth (GCB) consisted of 1.5% (w/v) special peptone, 0.4% (w/v) potassium phosphate dibasic (K_2_HPO_4_), 0.1% (w/v) potassium phosphate monobasic (KH_2_PO_4_) and 0.5% (w/v) sodium chloride (NaCl) with 0.043% NaHCO_3_. The antibiotic concentrations used for *E. coli* strains were as follows: ampicillin at 100 µg/ml, kanamycin at 50 µg/ml and chloramphenicol at 30 µg/ml. Those for *N. meningitidis* strains were as follows: kanamycin at 100 µg/ml, spectinomycin at 60 µg/ml, erythromycin at 3 µg/ml and tetracycline at 1 µg/ml. *E. coli* strains were transformed by chemical transformation [28]. Meningococci were transformed by the plate transformation procedure [29] or by chemical transformation. *E. coli* strain DH5α was used for the cloning and propagation of plasmids. Polymyxin minimum inhibitory concentrations (MICs) were determined using polymyxin B or E E-strips (bio Mérieux) as per manufacturer's instructions. For *E. coli* isolates, LB agar contained 0.4 mM isopropyl β-D-1-thiogalactopyranoside and was inoculated with 4×10^7^ colony forming units for each strain. A polymyxin-E E-strip was overlayed on the plate which was incubated overnight at 37°C and the zone of inhibition was read after 18 hrs. For meningococcal isolates, GCB was inoculated with 4×10^7^ colony forming units for each strain. A polymyxin-B E-strip was overlayed on the plate which was incubated overnight at 37°C and the zone of inhibition was read after 18 hrs.

### Construction of LptA::His_x6_ expression vectors for use in *E. coli* and *N. meningitidis*

All bacterial strains and plasmids used in this study are described in Table 1 and Table 2. For the expression of neisserial LptA::His_x6_ in *E. coli*, the high copy expression vector pCMK526 was engineered to contain *lptA* with a C-terminal His tag under control of the P*_Trc_* promoter. Wild-type *lptA* gene was PCR amplified from *N. meningitidis* strain NMB genomic DNA with primer pair KAP70 (5′-ATGTCTGCCGGACGTTTGAATGG-3′) and KAP71 (5′-ATTGCCGTGATCGGGAACTTGG-3′) and cloned into the low copy expression vector pHSG576 [30] to form pCMK519. *lptA* was PCR amplified from pCMK519 with primer pairs KAP96 (5′-AGCGGATAACAATTTCACACAGGA-3′) and KAP193 (5′-GGGAAGCTTTGCGCGGACGGCGGCAGGCTGCCAATATATC-3′), which replaced the stop codon with a *Hind*III site, then cloned into the *HincII* site of the low copy expression vector pHSG576 to form pCMK521. To enable directional cloning of *lptA* into pTrc99A and pCMK133, an *Nco*I site was included upstream of *lptA*. To do this, *lptA* gene was amplified from pCMK521 with KAP97 (5′-GTTTTCCCAGTCACGAC-3′) and KAP64 (5′-CATGCCATGGT*AGGAGG*TCCAA**ATG**ATAAAACCGAACCTGAGGCCGAAGC-3′) which introduces an *Nco*I site and *E. coli* consensus shine dalgarno site (italics) upstream of the *lptA* start codon (bold). The PCR product was treated with T4 DNA polymerase, ligated into the *HincII* site of pHSG576 to form pCMK522. To create a gene encoding LptA::His_x6_, *lptA* was cloned into the vector pCMK133, which contains a *Hin*dIII site for in frame fusion with a His_x6_-tag next to an *aphA-3* marker in the polylinker of pENTR4 (Invitrogen). The vector pCMK133 was engineered to enable the expression of a C-terminal His-tag protein when a gene of interest containing a *Hind*III site replacing the stop codon is cloned into the *Nco*I/*Hind*III site replacing *ccdB*. Both vector pCMK522 and pCMK133 were digested with *Nco*I and *Hind*III, ligated and transformed into *E. coli* strain DH5α. A clone that contained *lptA* within pCMK133 with a successful C-terminal His-tag fusion was named pCMK524. The *lptA::His_x6_* gene was excised from pCMK524 by restriction digest with *Nco*I and *Sma*I and ligated with pTrc99A [31] restricted with *Nco*I and *Sma*I to form pCMK526.

**Table 1:** Bacterial strains and plasmids used in this study.

Strain Name	Genotype	Major Phenotype	Minimal inhibitory concentration of polymyxin (µg/ml)	Reference
NMB	*B:2B:P1.2,5:L2 (CDC8201085)	DsbA1+, DsbA2+, DsbA3+, LptA+	384	[49]
CKNM101	NMBΔ*dsbA1::aadA*	DsbA1-, DsbA2+, DsbA3+, LptA+	128	[36]
CKNM102	NMBΔ*dsbA2::tetM*	DsbA1+, DsbA2-, DsbA3+, LptA+	128	[36]
CKNM105	NMBΔ*dsbA1::aadA*Δ*dsbA2::tetM*	DsbA1-, DsbA2-, DsbA3+, LptA+	64	[36]
CKNM204	NMBΔ*lptA::aadA*	DsbA1+, DsbA2+, DsbA3+, LptA-	0.38	This study
CKNM631	NMBΔ*dsbA3::aphA-3*	DsbA1+, DsbA2+, DsbA3-, LptA+	128	This study
CKNM210	NMBΔ*dsbA3::aphA-3* Δ*dsbA1::aadA*	DsbA1-, DsbA2+, DsbA3-, LptA+	128	This study
CKNM211	NMBΔ*dsbA3::aphA-3* Δ*dsbA2::tetM*	DsbA1+, DsbA2-, DsbA3-, LptA+	128	This study
CKNM212	NMBΔ*dsbA3::aphA-3* Δ*dsbA1::aadA* Δ*dsbA2::tetM*	DsbA1-, DsbA2-, DsbA3-, LptA+	32	This study
CKNM216	NMB expressing LptA::His_x6_ from shuttle vector pCMK1001	DsbA1+, DsbA2+, DsbA3+, LptA::His_x6_++	ND	This study
CKNM219	CKNM216 Δ*dsbA1::aadA*	DsbA1-, DsbA2+, DsbA3+, LptA::His_x6_++	ND	This study
CKNM221	CKNM219 Δ*dsbA2::tetM*	DsbA1-, DsbA2-, DsbA3+, LptA::His_x6_++	ND	This study
CKNM222	CKNM216 Δ*dsbA3::aphA-3*	DsbA1+, DsbA2+, DsbA3-, LptA::His_x6_++	ND	This study
CKNM755	CKNM212 transformed with pCMK946 expressing LptA::His_x6_	DsbA1-, DsbA2-, DsbA3-, LptA::His_x6_++	ND	This study
*E. coli* DH5α	*fhuA2 lac(del)U169 phoA glnV44 Φ80' lacZ(del)M15 gyrA96 recA1 relA1 endA1 thi-1 hsdR17*	Chromosomal EcDsbA+	ND	[50]
*E. coli* JM109	JM107 *recA1*	Chromosomal EcDsbA+	0.094	[51]
JCB570	MC1000 *phoR zih12*::Tn10	Chromosomal EcDsbA+	0.094	[34]
JCB571	*dsbA* null mutant of JCB570	Chromosomal EcDsbA-	0.094	[34]
EXEC94	JM109 containing pTrc99A	Chromosomal EcDsbA+	0.094	This study
CKEC272	JCB571 expressing EcDsbA from pCMK255	EcDsbA+++ (chromosomal and plasmid EcDsbA)	0.094	[35]
CKEC288	JCB571 carrying pTrc99A	Chromosomal EcDsbA+	ND	
CKEC526	JM109 expressing LptA::His_x6_ from pCMK526	Chromosomal EcDsbA+ and LptA::His_x6_++	1.5	This study
CKEC543	JCB571 expressing LptA::His_x6_ from pCMK526	EcDsbA-, LptA::His_x6_++	0.094	This study
CKEC564	JCB571 expressing LptA::His_x6_ from pCMK526 and EcDsbA from pCMK255	Plasmid EcDsbA++ and LptA::His_x6_++	3.0	This study
CKEC585	JM109 expressing LptA::His_x6_ from pCMK526 and EcDsbA from pCMK255	EcDsbA+++ (chromosomal and plasmid EcDsbA) and LptA::His_x6_++	3.0	This study

*Nomenclature is derived from serological typing scheme for capsule polysaccharide (serogroup B):porin B variant (2B):porin A variant (P1.2,5):lipooligosaccharide immunotype (L2). Cassettes: *aadA*  =  spectinomycin resistance, *tetM*  =  tetracycline resistance, *aphA-3*  =  kanamycin resistance, *ermC*  =  erythromycin resistance.

+ ND =  not done.

**Table 2:** Plasmids used in this study.

Plasmid Name	Description*	Reference
pYT250	High copy neisserial shuttle vector	[15]
pHSG576	Low copy cloning vector (chloramphenicol resistant)	[30]
pUC18K	pUC18 carrying *aphA-3* non-polar cassette (ampicillin and kanamycin resistant)	[32]
pTrc99A	High copy expression vector (ampicillin resistant)	[31]
pJSK411::*ompR*	Vector containing *ompR* promoter driving the expression of GFP	[33]
pKA314	Vector containing *lptA::aadA* knockout cassette	[13]
pJKD2639	pHSG576 carrying *dsbA1::aadA* knockout cassette	[36]
pJKD2641	pHSG576 carrying *dsbA2::tetM* knockout cassette	[36]
pJKD2643	pHSG576 carrying *dsbA3::ermC* knockout cassette	[36]
pCMK133	pENTR4 containing a modified polylinker for the fusion of a His_x6_-tag with an open reading frame terminating in *Hin*dIII	[38]
pCMK255	pHSG576 expressing EcDsbA	[35]
pCMK519	pHSG576 carrying *lptA* amplified with primer pair KAP70 and KAP71	This study
pCMK521	pHSG576 carrying *lptA* with the stop codon replaced by *Hin*dIII in the *Hinc*II site	This study
pCMK522	pHSG576 carrying the Shine Dalgarno site-*lptA*-*Hin*dIII in the *Hinc*II site	This study
pCMK524	pCMK133 containing *lptA*::*His_x6_* cloned into the *Nco*I and *Hin*dIII site	This study
pCMK526	pTrc99A containing *lptA*::*His_x6_* cloned into the *Nco*I and *Sma*I site	This study
pCMK597	pHSG576 containing the *aphA-3* cassette from pUC18K cloned into the *Sca*I and *Msc*I sites of *cat1*.	This study
pCMK598	pCMK596 containing a re-engineered multiple cloning site formed by KAP584	This study
pCMK599	pCMK598 containing the GC cryptic plasmid from pYT250 cloned into the *Hin*dIII site	This study
pCMK600	pCMK599 containing the *ompR* promoter cloned into the *Bam*HI/*Eco*RI site.	This study
pCMK630	pHSG576 containing an internal fragment of *dsbA3* with introduced internal *Kpn*I and *Xba*I sites	This study
pCMK631	pCMK630 containing *aphA-3* cloned into the internal *Kpn*I and *Xba*I sites to create *dsbA3::aphA-3*	This study
pCMK940	pHSG576 containing the GC-cryptic plasmid from pYT250 cloned into the *Hin*dIII site.	This study
pCMK946	pCMK940 containing *lptA::His_x6_* under the control of P_ompR_ cloned into the *Eco*RI and *Sma*I sites	This study
pCMK1001	pCMK600 containing *lptA::His_x6_* under the control of P*_ompR_*	This study

*Cassettes: *aadA*  =  spectinomycin resistance, *tetM*  =  tetracycline resistance, *aphA-3*  =  kanamycin resistance, *ermC*  =  erythromycin resistance.

For the expression of neisserial LptA::His_x6_ in *N. meningitidis*, the low copy expression vector pHSG576 [30] was modified to contain the GC cryptic backbone of pYT250 [15] to allow replication in *Neisseria*, to confer resistance to kanamycin instead of resistance to chloramphenicol and to contain *lptA* downstream of the neisserial P*_ompR_* promoter. Briefly, the *aphA-3* gene was liberated from the vector pUC18K [32] by restriction digest with *Sma*I and cloned into *Sca*I and *Msc*I disgested pHSG576 [30] to disrupt *cat*1 and form pCMK597. A 72 bp multiple cloning site (MCS) was engineered. Briefly, the primers KAP584 (5′-GAATTCGGATCCACAGGAGGCAGACCATGGCCACTCGAGAGCTCGGTACCCGGGCATGCATCTAGAGTCGAC-3′) and KAP585 (5′-GTCGACTCTAGATGCATGCCCGGGTACCGAGCTCTCGAGTGGCCATGGTCTGCCTCCTGTGGATCCGAATTC-3′) were resuspended in 1×T4 DNA ligase buffer (New England Biolabs) at equimolar concentrations and annealed by incubation at 90°C for 5 minutes followed by incubation at room temperature for 60 minutes. The resultant fragment was restricted with *Eco*RI and *Hinc*II and cloned into pCMK597, restricted with the same enzymes, to form pCMK598. The GC cryptic backbone from pYT250 was excised by restriction digest with *Hin*dIII and cloned into the *Hin*dIII site of pCMK598 to form the neisserial shuttle vector pCMK599. The neisserial P*_ompR_* promoter was liberated from pJSK411::*ompR* [33] by digest with *Bam*HI and *Eco*RI and cloned into the *Bam*HI and *Eco*RI restriction sites of the MCS of pCMK599 to form pCMK600. The *lptA*::His_x6_ construct was liberated from pCMK526 by restriction digest with *Nco*I and *Sma*I and cloned into the *Nco*I and *Sma*I restriction sites of the MCS of pCMK600 to form the neisserial LptA::His_x6_ expression vector, pCMK1001. To create an expression shuttle vector with a chloramphenicol marker, the GC cryptic plasmid from pYT250 was cloned into the *Hin*dIII site of pHSG576 to create pCMK940. The P*_ompR_*-*lptA*::His_x6_ cassette was excised from pCMK1001 with the restriction sites *Sma*I and *Eco*RI and ligated into these sites in pCMK940 to create pCMK946.

### Construction of LptA::His_x6_ and oxidoreductase expressing *E. coli*

The wild-type *E. coli* strain JCB570 and corresponding *E. coliΔdsbA* mutant strain JCB571 were used as control strains [34]. *Ec*DsbA was expressed from pCMK255 as described previously [35]. To complement JCB571 with *Ec*DsbA, pCMK255 was transformed into this host to create CKEC272 [35]. To construct an *E. coli* strain expressing LptA::His_x6_ without any oxidoreductase, JCB571 was transformed with pCMK526 to form CKEC543. To create an *E. coli* strain co-expressing LptA::His_x6_ and *Ec*DsbA, CKEC543 was transformed with pCMK255 to form CKEC564.

### Construction of LptA and oxidoreductase mutants in *N. meningitidis*

To make a *ΔlptA::aadA* mutant in strain NMB, pKA314 [13] was transformed into strain NMB and was stored as CKNM204. CKNM101 (NMBΔ*dsbA1*), CKNM102 (NMBΔ*dsbA2*) and CKNM105 (NMBΔ*dsbA1*/*dsbA2*) were created as described in Kumar *et. al.* [36]. CKNM631 (NMBΔ*dsbA3*) was constructed by transformation of pCMK631 (pHSG576 containing *dsbA3::aphA-3*) into *N. meningitidis* strain NMB. The vector pCMK631 was constructed by SOE PCR to introduce an internal *Kpn*I and *Xba*I into which an *aphA-3* cassette was inserted. In the first round of SOE PCR, an internal region of *dsbA3* from NMB genomic DNA was amplified with primer pairs DAP267 with KAP415 (5′-TCTAGAGGTACCGCCAGACCACGTGCTCCGTCC-5) and DAP265 with KAP416 (5′-GGTACCTCTAGAAGCCTGAAATGCTCGGTCTGG-3′). In the second round of SOE PCR, the two fragments from the previous reaction were used as template for amplification using KAP418 (5′-GCTGTCGGCAGTGTTGTCCGC-3′) and KAP419 (5′-GGAGAGTCGTAGGCGCGCATC-3′). This fragment was cloned into the *Hinc*II site of pHSG576, resulting in pCMK630. The gene *aphA-3* conferring kanamycin resistance was liberated from pUC18K by *Kpn*I and *Xba*I, then cloned into the *Kpn*I and *Xba*I sites of pCMK630 resulting in pCMK631. CKNM631 was transformed with pJKD2639 and pJKD2641 [36] to form strains CKNM210 (NMBΔ*dsbA1/dsbA3*) and CKNM211 (NMBΔ*dsbA2/dsbA3*), respectively. CKNM210 was transformed with pJKD2641 [36] to form the triple knockout strain CKNM212 (NMBΔ*dsbA1*/*dsbA2/dsbA3*) and incubated at 30°C with 5% CO_2_.

### Expression of LptA::His_x6_ in oxidoreductase mutants of *N. meningitidis.*

The neisserial LptA::His_x6_ expression vector pCMK1001 was transformed into *N. meningitidis* strain NMB to form strain CKNM216. CKNM219 (CKNM216Δ*dsbA1*) was constructed by transformation of pCMK109 [36] into CKNM216. CKNM221 (CKNM216Δ*dsbA1*/*dsbA2*) was constructed by transformation of the vector pJKD2641 [36] into CKNM219. CKNM222 (CKNM216Δ*dsbA3*) was constructed by chemical transformation of the vector pJKD2643 [36] into CKNM216. To create a neisserial oxidoreductase triple mutant expressing LptA::His_x6_, pCMK946 was naturally transformed into CKNM212. The retention of the plasmid was confirmed by extraction of the plasmid and restriction mapping, followed by a Western immunoblot which confirmed that LptA::His_x6_ was expressed.

### Purification of LptA::His_x6_ from CKNM216

The membrane fraction of CKNM216 was separated by resuspending the growth from four overnight plates in 30 ml binding buffer (20 mM Na_3_PO_4_, 500 mM NaCl, 20 mM imidazole, pH 7.4) and sonicated on ice for 30 mins (30 sec on, 30 sec off). The suspension was centrifuged at 3000×g, 20 mins and the supernatant further centrifuged at 100,000×g for 120 mins at 4°C. The supernatant was discarded and the pellet re-suspended in 20 ml binding buffer containing 2% triton X-100 at room temperature for 3 hrs. The suspension was centrifuged at 50,000×g for 30 mins at 4°C. LptA::His_x6_ was purified from the supernatant on a HisTrap FF column (GE Helathcare Life Sciences). Following equilibration and application of the supernatant, the column was washed with 10 volumes of binding buffer containing 2% triton X-100 and the protein eluted in 10 volumes of elution buffer (20 mM sodium phosphate, 0.5 M NaCl and 500 mM imidazole, pH 7.4) containing 2% triton X-100 as per manufacturer′s instructions. The eluate was dialysed three times against 10% glycerol, 20 mM Tris-HCl, pH 7.9), and the protein concentrated to 0.5 ml using a Centricon 10 column as per manufacturer′s instructions (Millipore).

Detection of the expression of LptA::His_x6_ was determined by western immunoblotting. Whole cell lysates (1 µg) were separated by 4% stacking and 12% separating sodium dodecyl sulfate-polyacrylamide gel electrophoresis (SDS-PAGE) by standard methods and transferred to nitrocellulose membranes. The membranes were blocked for 1 hr at room temperature with 2% BSA in TBS. The polyclonal rabbit anti-LptA IgG [19] and the conjugated monoclonal mouse anti-His-HRP (Sigma) primary antibodies were used at 1∶500 and 1∶1,000 in blocking buffer respectively, and the membranes incubated overnight at room temperature. Horse radish peroxidase-conjugated anti-rabbit IgG secondary antibody (Santa Cruz Biotechnology) was used for detection of rabbit anti-LptA IgG at a concentration of 1∶1,000 in blocking buffer for 3 hrs at room temperature and the membranes were developed by colormetric analysis using with 30 mg 4-chloro-naphthol (Sigma) dissolved in 10 ml methanol plus 30 µl H_2_O_2_ (Univar) in 40 ml Tris-NaCl, pH 7.4.

### Determination of the redox status of LptA::His_x6_

The redox states of LptA::His_x6_ *in vivo* were determined by the same method as described previously for DsbA [37]. Briefly, growth from overnight plates was resuspended in broth to an OD_600_ = 1.2 for *E. coli* cultures, and an OD_560_ = 2.0 for *N. meningitidis* cultures. Trichloroacetic acid (final concentration 5% v/v) was added directly to 1 ml of culture to denature and precipitate whole-cell proteins, which were then collected by centrifugation, washed with acetone and dissolved in 100 µl 1% SDS–100 mM Tris–HCl (pH 7.5) containing 20 mM 4-acetamido-4′-maleimidylstilbene-2,2′-disulfonic acid (AMS). For a reduced control, 1 ml of culture was treated with 17 mM dithiothreitol (DTT) for 10 minutes at 37°C with shaking prior to treatment with TCA (5% v/v) and AMS alkylation as described above. In addition, 1 ml of culture was centrifuged and resuspended in 100 µl sterile H_2_O. All samples were boiled for 10 minutes after the addition of 25 µl of 5× sample buffer without reducing agent. Samples were loaded onto a 4% stacking/12.5% bis/acrylamide SDS–PAGE which was run at 100 V in the cold room. The proteins were transferred to a Hybond C nitrocellulose membrane (Amersham Life Sciences) at 100 V for 1.5 hr using a Bio-Rad PROTEAN 3 Western blot apparatus (Bio-Rad Laboratories). The membrane was probed with mouse anti-His_x6_ antibody (Sigma) diluted 1∶1000 in blocking buffer (2% BSA in Tris-NaCl, pH 7.4), incubated at 37°C with shaking, for 3–4 hours. The membrane was then probed with the HRP-linked donkey anti-mouse IgG (Santa Cruz Biotechnology) diluted 1∶1000 in blocking buffer and incubated at 37°C for 2 hrs with shaking. The membranes were developed with either the ECL detection kit Western Blotting Analysis System (Amersham Life Sciences) or by colorimetric solutions as described previously. Purified truncated *Nm*LptAΔMA which migrates as a 31 kDa protein was prepared according to Anandan *et al*. [38].

### Purification of LOS/LPS and analysis by mass spectroscopy

The LOS/LPSs were prepared by hot phenol/water extraction [39]. The crude LOS/LPS from the aqueous phase was dialyzed against deionized water using the aqueous phase 1000 MWCO dialysis tubing. After dialysis, the samples were freeze-dried and resuspended in a solution of 20 mM Tris-HCl and 2 mM MgCl_2_ at pH 8.0. DNase I (100 µl of 7 mg/ml in 20 mM Tris-HCl, pH 8.0), and RNase A (100 µl of 17 mg/ml) were added and the solution was incubated for 3 hrs at 37°C. After adding 400 µg of proteinase K and adding CaCl_2_ to a final concentration of 2 mM, the sample was incubated at 37°C overnight. The LOS was pelleted by ultracentrifugation at 100,000×g for 18 hrs. The pellets were re-suspended in water and re-centrifuged at 100,000×g for 4 hrs. The resulting pellets were suspended in water, freeze-dried analysed as described.

The various lipid A samples were prepared by subjecting each LOS preparation to 1% SDS in 10 mM sodium acetate buffer, pH 4.5 at 100°C for 1 hr, followed by lyophilisation. The SDS was removed by washing the dried residue with 100 µl of distilled water and 500 µl of acidified ethanol (prepared by combining 100 µl of 4 M HCl with 20 ml of 95% ethanol), followed by centrifugation (3,000×g, 20 min). The precipitate was then washed with 500 µl of 95% ethanol and centrifuged. The centrifugation and washing steps were then repeated. The final precipitate was lyophilized to give purified lipid A. Lipid A fractions were analysed by matrix assisted laser desorption ionization time of flight mass spectrometry (MALDI-TOF-MS) using an AB SCIEX TOF/TOF 5800 (Applied Biosystems). Lipid A samples were dissolved in 3∶1 chloroform:methanol solution and mixed with 0.5 M 2,4,6-trihydroxyacetophenone (THAP) matrix in methanol in a 1∶1 ratio. Mass calibration was performed with Angiotensin I (*m/z* 1296·69), Glu-fibrinopeptide B (*m/z* 1570·68), ACTH (18–39 clip, *m/z* 2465·20) and ACTH (7–38 clip, *m/z* 3657·93). Analysis of the lipid A samples was performed in the negative reflector mode.

## Results

### Expression of neisserial LptA in *E. coli* increases resistance to polymyxin and is dependent upon the co-expression of an oxidoreductase

To examine whether neisserial LptA stability was dependent upon oxidoreductases, we first established a model to detect neisserial LptA expression in *E. coli* (Table 1). Expression of LptA::His_x6_ in *E. coli* JM109 (CKEC526, 1.5 µg/ml) resulted in a 16-fold increase in polymyxin MIC when compared to JM109 carrying the empty vector (EXEC94, 0.094 µg/ml). To test for the dependency of neisserial LptA stability on the presence of an oxidoreductase, LptA::His_x6_ was expressed in JCB571 (CKEC543) in which the chromosomal copy of *dsbA* had been insertionally inactivated [34]. Expression of LptA in *E coli* lacking a functional DsbA had no effect on polymyxin resistance and the MIC (0.094 µg/ml) was the same in both CKEC543 and JCB571. When JCB571 was complemented with a vector expressing *Ec*DsbA (CKEC272), no difference in MIC from the parental wild-type JCB571 was detected. However, co-expression of neisserial LptA::His_x6_ and *Ec*DsbA in JCB571 (CKEC564) resulted in an increase of polymyxin MIC to 3 µg/ml, representing a 32-fold increase in MIC over parental wild-type JCB571. Therefore, neisserial LptA::His_x6_ increases resistance of *E. coli* to polymyxin only when expressed in the presence of an oxidoreductase.

### LptA catalyses the addition of PEA groups to *E. coli* lipid A

**Figure 1:**
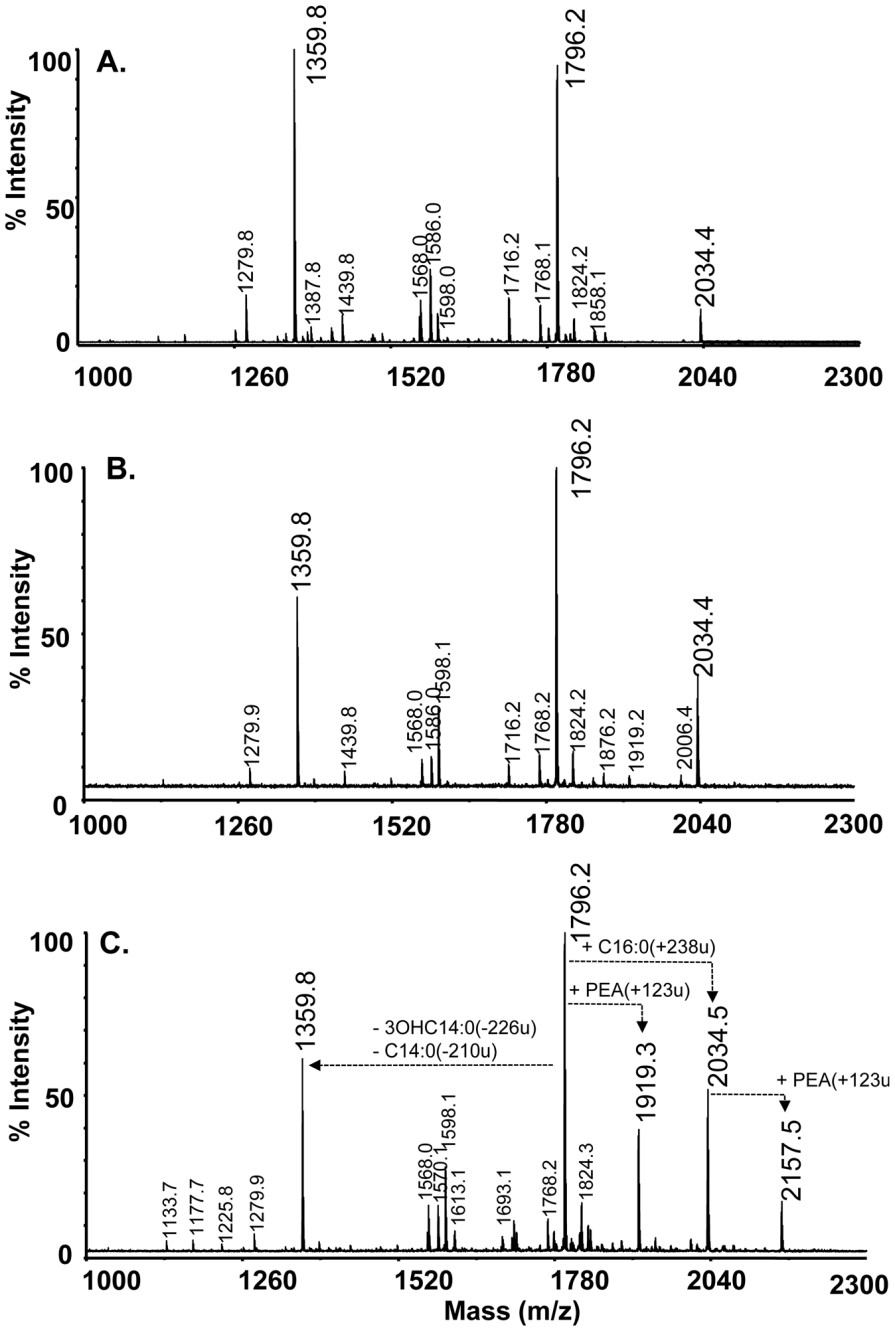
Neisserial LptA::His_x6_ transfers PEA to lipid A of *E. coli* LPS. Lipid A profiles of LPS extracted from *E. coli* strains JCB571 expressing *Ec*DsbA (CKEC272) (Panel A), *E. coli* JCB571 expressing LptA::His_x6_ (CKEC543) (Panel B) and JCB571 expressing LptA::His_x6_ and *Ec*DsbA (CKEC564) (Panel C) as determined by MALDI-TOF MS. *bis*-Phosphorylated hexaacylated lipid A (m/z = 1796), the mono-phosphorylated derivative (*m/z* = 1716), and the heptaacylated version due to the addition of a palmitic acyl residue (*m/z* = 2034) were detected in all strains. *bis*-Phosphorylated tetraacylated lipid A (*m/z* = 1360) was found abundantly in the MALDI spectra of all three strains, which was likely produced from *bis*-phosphorylated hexaacylated lipid A (*m/z* = 1796) during the ionization step on MALDI. The lipid A preparations from CKEC543 expressing LptA (Panel B) and CKEC564 co-expressing LptA and *Ec*DsbA (Panel C) also contained ions consistent with one PEA added to the *bis*-phosphorylated structure (such as *m/z* 1919; i.e. 1796+123) and the heptaacylated structure (such as *m/z* = 2157, i.e. 2034+123).

To determine the effects of LptA::His_x6_ expression on modifications to lipid A in *E. coli*, samples of lipid A were prepared from *E. coliΔdsbA* JCB571 strains expressing LptA::His_x6_ and/or *Ec*DsbA and analysed by mass spectrometry (see methods). MALDI-TOF MS analysis of the various lipid A samples is shown in Figure 1. The lipid A from strains *E. coliΔdsbA* JCB571 and JCB570 (wild-type parent) gave the same spectra as that shown for the lipid A from JCB571 carrying a vector expressing *Ec*DsbA (CKEC272). The observed ions were consistent with the normal *E. coli* lipid A molecules; namely the *bis*-phosphorylated hexaacylated structure (*m/z* = 1796), its mono-phosphorylated derivative (*m/z* = 1716), and its heptaacylated version due to the addition of a palmitic acid residue (*m/z* = 2034). The lipid A preparations from strains JCB571 carrying LptA::His_x6_ (CKEC543) and JCB571 co-expressing LptA::His_x6_ and *Ec*DsbA (CKEC564) showed these ions plus additional ions due to the addition of PEA to the mono-phosphorylated structure (*m/z* 1839, i.e. 1716+123) and the *bis*-phosphorylated structure (*m/z* 1919; i.e. 1796+123). In addition, some minor lipid A molecules with a single PEA (*m/z* 1693, 1891, 1947, and 2157) were also detected along with those without PEA (*m/z* 1570, 1768, 1824 and 2034) in both CKEC543 and CKEC564. However, the ratio of total peak area of the lipid A molecules with PEA (i.e. the peak area of the *m/z* 1839+1919+1693+1891+1947+2157 ions) to those without PEA (i.e. the peak area of the *m/z* 1716+1796+1570+1768+1824+2034 ions) was ten-fold less in strain JCB571 with LptA::His_x6_ alone than for JCB571 co-expressing LptA::His_x6_ and *Ec*DsbA (0.036 versus 0.36). Thus, a single PEA addition to lipid A of LPS of JCB571 strains expressing LptA::His_x6_ (CKEC543 and CKEC564) was detected that is not present in control strains JCB570 (wild type), JCB571 (*ΔdsbA*) and JCB571 expressing *Ec*DsbA alone (Figure 1). This addition is more prevalent in JCB571 co-expressing LptA::His_x6_ and *Ec*DsbA than in JCB571 with LptA::His_x6_ alone and correlates with increased resistance to polymyxin of the former strain. This experiment confirms that neisserial LptA::His_x6_ is able to transfer PEA to lipid A of *E. coli* LPS resulting in increased resistance to polymyxin when co-expressed with the oxidoreductase *Ec*DsbA.

### The stability of neisserial LptA::His_x6_ is dependent upon the presence of *Ec*DsbA in an *E. coli* host

**Figure 2:**
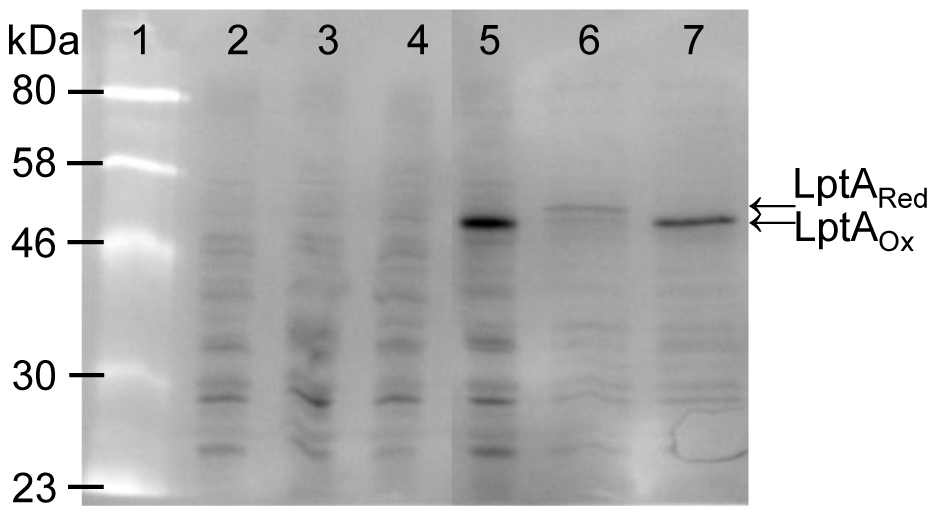
LptA::His_x6_ stability is dependent upon oxidoreductase activity in *E. coli*. Standardised whole cell lysates were separated by SDS-PAGE. A Western immunoblot was developed using anti-His tag antibody to detect the presence of LptA::His_x6_ in the cellular extracts. Lanes were: Lane 1, ColorPlus pre-stained protein molecular weight marker (New England Biolabs); Lane 2: *E. coli* JCB571 expressing *Ec*DsbA (CKEC272); Lane 3: *E. coli* JCB571 carrying pTrc99A (CKEC288); Lane 4: *E. coli* JCB571 expressing LptA::His_x6_ (CKEC543); Lane 5: *E. coli* JCB571 expressing LptA::His_x6_ and *Ec*DsbA (CKEC564); Lane 6: CKEC564 treated with DTT and alkylated with AMS; and Lane 7: CKEC564 alkylated with AMS. Molecular weights (kDa) are indicated on the left.

Alkylation with 4-acetamido-4′-maleimidylstilbene-2,2′-disulfonic acid (AMS) is a technique used to determine the *in vivo* redox states of proteins [40]. AMS forms covalent adducts with free thiol groups of reduced cysteine residues adding ∼500 a.m.u. to the mass of a protein for each reduced cysteine residue modified which can be detected by mobility shift on SDS-PAGE. Therefore, an immunoblot using anti-His_x6_ antibody was used for the detection of LptA::His_x6_ in AMS alkylated and untreated cell lysates from *E. coli* JCB571 co-expressing LptA::His_x6_ and EcDsbA (CKEC564). Isolates JCB571, JCB571 containing pTrc99A (CKEC288), JCB571 expressing *Ec*DsbA, alone and JCB571 expressing LptA::His_x6_ alone were used as controls. A protein of molecular weight of 48 kDa was detected in JCB571 co-expressing LptA::His_x6_ and EcDsbA but not in any other strain (Figure 2). Intact LptA is predicted to have a molecular weight of 60 kDa. A comparison of the whole cell lysates with the purified LptA::His_x6_ which has been shown to be intact by crystallisation (data not shown) indicated that the protein in the lysates was the same mass as the purified sample (Figure S1). Thus it appears that the stained molecular weight markers were inaccurate as a standard for determining mass under the conditions used for these SDS-PAGE gels.

In the absence of oxidoreductases, proteins without disulphide bonds will either accumulate in the periplasm as reduced proteins, or will be more sensitive to proteolytic degradation and be removed entirely. Since LptA::His_x6_ expression was detected in JCB571 in which *Ec*DsbA was also present but not where LptA::His_x6_ is expressed in the absence of a functional oxidoreductase, we hypothesised that DsbA catalysed disulfide bond formation in LptA::His_x6_ was required for stability of LptA. In the absence of DsbA, LptA::His_x6_ would become sensitive to proteolytic degradation in the periplasm and therefore was absent in JCB571 expressing LptA::His_x6_ alone. This hypothesis is supported by the observation that LptA::His_x6_ was partially degraded (fainter band) in the presence of EcDsbA when the cell lysate was pre-treated with the reducing agent DTT which would have reduced the disulphide bonds in periplasmic proteins (Figure 2). Finally, AMS alkylation of JCB571 co-expressing LptA::His_x6_ and *Ec*DsbA did not result in a mobility shift in the size of LptA::His_x6_. This result indicates that there were no free thiol groups for alkylation in LptA::His_x6_ because it was oxidised by *Ec*DsbA. In conclusion, LptA::His_x6_ contains *Ec*DsbA catalysed disulfide bonds that are necessary for protein stability in the periplasm. In the absence of *Ec*DsbA, LptA::His_x6_ is rapidly removed via proteolytic degradation.

### All three neisserial oxidoreductases contribute to resistance to polymyxin

Unlike *E. coli*, which possesses a single oxidoreductase, *Neisseria* spp. contain up to three oxidoreductases, termed DsbA1, DsbA2 and DsbA3. To examine whether any or all of the neisserial oxidoreductases were required for the stability of LptA in neisseria, we examined the polymyxin resistance profile of a series of meningococcal oxidoreductase mutants. *N. meningitidis* strain NMB contains all three oxidoreductases. The MIC of wild-type strain NMB to polymyxin is 384 µg/ml while NMBΔ*lptA* is 1000-fold more sensitive with an MIC of 0.38 µg/ml. Mutation of each of the individual oxidoreductases in turn resulted in a 3-fold decease in MIC to 128 µg/ml. All combinations of double mutations were tested and only a double mutation in both *dsbA1* and *dsbA2* resulted in a further 2-fold decrease in MIC to 64 µg/ml (Table 1). However, since the level of polymyxin resistance of the NMBΔ*dsbA1/dsbA2* double mutant was still 164-fold above the level obtained for the Δ*lptA* mutant, the NMBΔ*dsbA1*/*dsbA2/dsbA3* triple mutant was examined. Since the NMBΔ*dsbA1*/*dsbA2/dsbA3* triple mutant is temperature sensitive, all of the strains were compared at 30°C. The MICs for the single and double mutants remained unchanged at 30°C (data not shown). Interestingly, the NMBΔ*dsbA1*/*dsbA2/dsbA3* triple mutant was sensitive to a MIC of 32 µg/ml of polymyxin (Table 1) which is 2-fold less than that of NMBΔ*dsbA1/dsbA2* but still 84-fold greater than the MIC for the NMBΔ*lptA* mutant.

### LptA is reduced but remains stable in the absence of disulphide bonds in the neisserial periplasm

**Figure 3:**
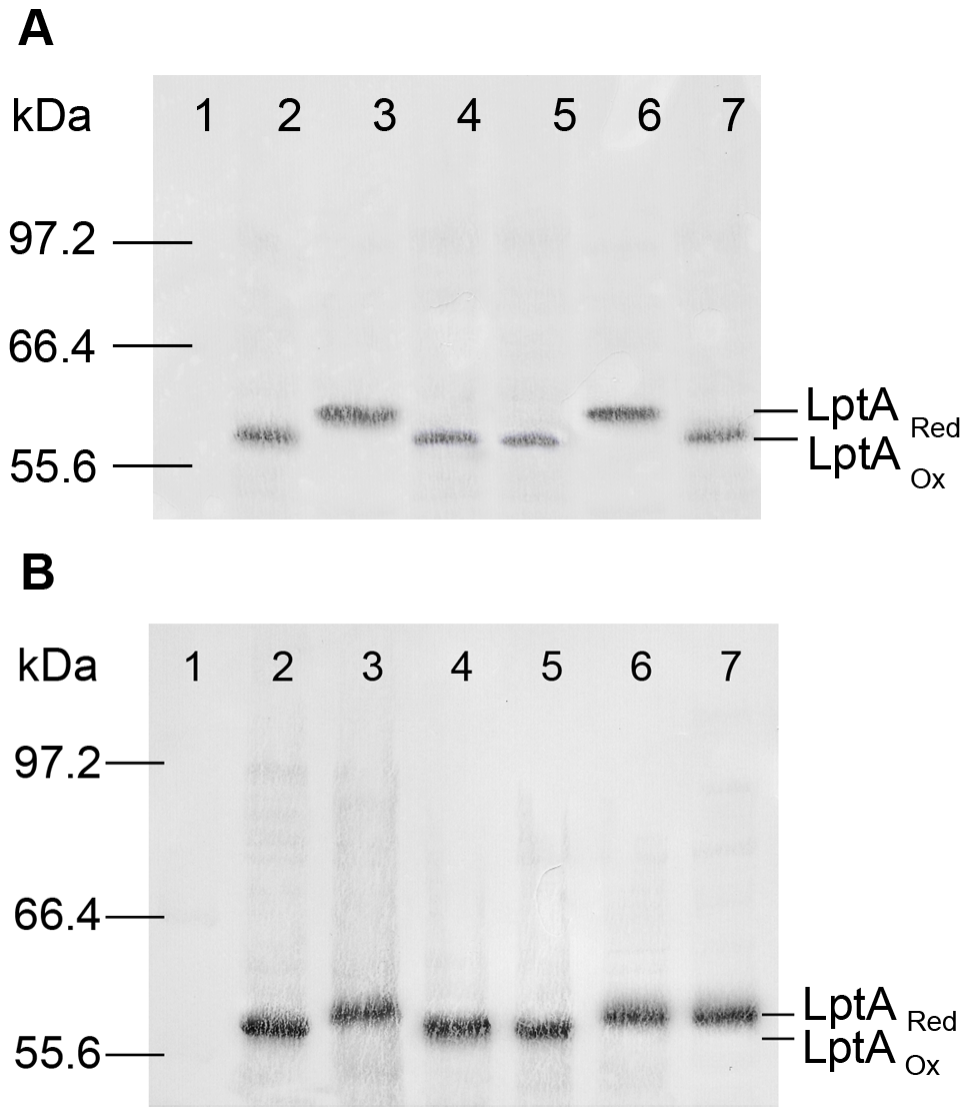
Oxidation status of LptA::His_x6_ in oxidoreductase mutants of *N. meningitidis*. Standardised cell lysates were separated by SDS-PAGE, followed by transfer to a membrane and western immunoblot using anti-His_x6_ HRP conjugate antibody to detect the presence of LptA::His_x6_. Panel A. Lane 1, protein molecular weight standard (New England Biolabs, Cat-2-212); Lane 2: NMBΔ*dsbA1/dsbA2* expressing LptA::His_x6_ from pCMK1001 (CKNM221) untreated; Lane 3: CKNM221 treated with DTT and alkylated with AMS; Lane 4: CKNM221 alkylated with AMS, Lane 5: NMBΔ*dsbA3* expressing LptA::His_x6_ (CKNM222) untreated; Lane 6: CKNM222 treated with DTT and alkylated with AMS; Lane 7: CKNM222 alkylated with AMS. Panel B. Lane 1, protein molecular weight standard (New England Biolabs, Cat-2-212); Lane 2: NMB expressing LptA::His_x6_ (CKNM216) untreated; Lane 3: CKNM216 treated with DTT and alkylated with AMS; Lane 4: CKNM216 alkylated with AMS; Lane 5: NMBΔ*dsbA1*/*NmdsbA2/dsbA3* expressing LptA::His_x6_ (CKNM755); Lane 6: CKNM755 treated with DTT and alkylated; Lane 7: CKNM755 alkylated with AMS.

To test the redox status of LptA in *N. meningitidis*, LptA::His_x6_ was expressed from a neisserial shuttle vector pCMK964 (chloramphenicol resistant) or pCMK1001 (kanamycin resistant) in a variety of mutant backgrounds in which different combinations of oxidoreductases were removed by insertional inactivation of the chromosomal loci. To confirm that the LptA::His_x6_ was expressed and intact in *N. meningitidis* hosts, whole cell lysates of *N. meningitidis* strain NMB carrying pCMK1001 (CKNM216) were compared to whole cell lysates prepared from NMBΔ*lptA, E. coli* JCB571 co-expressing LptA::His_x6_ with EcDsbA, and *E. coli* JCB571 expressing EcDsbA alone. Immunoblots using anti-His-tag antibody (Figure S2, Panel A) and anti-LptA antibody (Figure S2, Panel B) detected a protein of the correct mass which was consistent in size with the protein detected in the *E. coli* cell lysates. To confirm that *lptA::His_x6_* gene was intact, pCMK1001 was extracted from strain NMB and fully sequenced. In addition, LptA::His_x6_ was purified using the nickel columns from preparations of the neisseria membrane and was shown to react with both the anti-His-tag and anti-LptA antibodies (Figure S2). To determine the effect of oxidoreductase expression on LptA::His_x6_ stability, pCMK1001 was introduced into the oxidoreducatase mutant backgrounds. Whole cell lysates of these strains were alkylated, separated by SDS-PAGE, transferred to a membrane and immunobloted using an anti-His HRP conjugate antibody to detect LptA::His_x6_ (Figure 3). LptA::His_x6_ is oxidised in each of the alkylated samples from NMBΔ*dsbA1* expressing LptA::His_x6_ (CKNM219), NMBΔ*dsbA1*/*dsbA2* expressing LptA::His_x6_ (CKNM221) and NMBΔ*dsbA3* expressing LptA::His_x6_ (CKNM222). Strains expressing either DsbA1 or DsbA2 alone also produced oxidised LptA::His_x6_ (data not shown). However, in NMBΔ*dsbA1*/*dsbA2*/*dsbA3* expressing LptA::His_x6_ (CKNM755) reduced LptA::His_x6_ accumulated. In conclusion, these alkylating assays indicate that DsbA1, DsbA2, and DsbA3 donate disulphide bonds to LptA. In addition, since reduced LptA::His_x6_ accumulated in the triple oxidoreductase mutant, this protein remained stable and was not immediately degraded in the absence of disulphide bonds.

### The neisserial oxidoreductase, DsbA3, contributes to the activity of LptA

**Figure 4:**
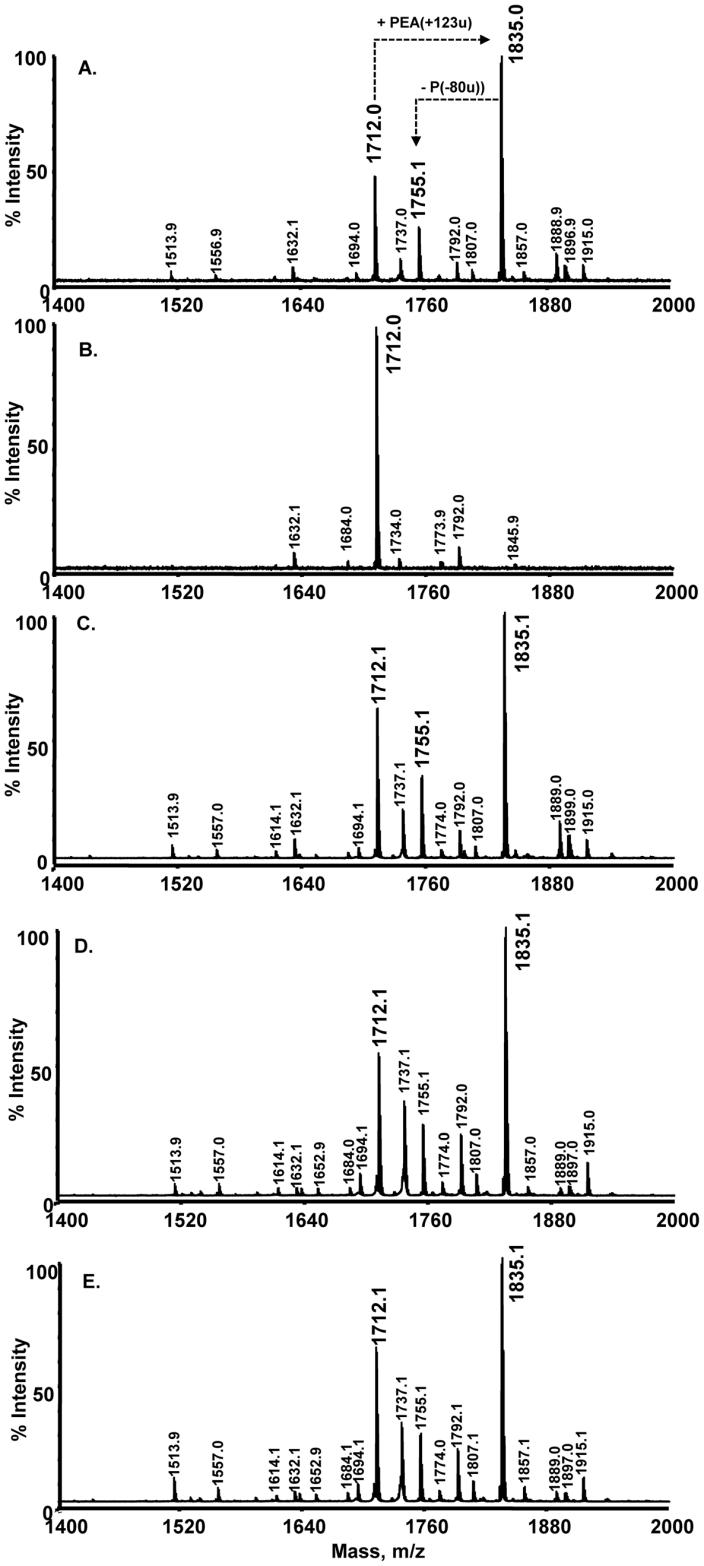
Lipid A substitution profiles of meningococcal oxidoreductase mutants. Lipid A profiles of LOS extracted from *N. meningitidis* strain NMB (Panel A), NMBΔ*lptA::aadA* (Panel B), NMBΔ*NmdsbA1*/*NmdsbA2* (Panel C), NMBΔ*NmdsbA3* (Panel D) and NMBΔ*dsbA1/dsbA2/dsbA3* (Panel E) as determined by MALDI-TOF MS. *bis*-Phosphorylated hexaacylated lipid A (*m/z* = 1712), the *mono*-phosphorylated (*m/z* = 1632) and the *tri*-phosphorylated derivative (*m/z* = 1792) were detected in all strains. Strain NMB and the oxidoreductase mutants all expressed the mono-phosphorylated, *bis*-phosphorylated and *tri*-phosphorylated hexaacylated lipid A with a single PEA addition (*m/z* = 1755, *m/z* = 1835 and *m/z* = 1915). Consistent with the loss of LptA activity, NMBΔ*lptA::aadA* lacked these ions.

Although mutation of the oxidoreductases resulted in decreased resistance to polymyxin in neisseria, it was not certain whether this phenotype correlated with a change in the substitution profile of the lipid A with PEA headgroups in the oxidoreductase mutants. To examine this further, the PEA substitution profile of lipid A was examined by MS from the oxidoreductase mutants and NMBΔ*lptA* (Figure 4). The lipid A preparations from oxidoreductase mutants and the control wild-type strain NMB possessed ions consistent with the addition of PEA to the mono-phosphorylated structure (*m/z* = 1755, i.e. 1632+123), the *bis*-phosphorylated structure (*m/z = *1835; i.e. 1712+123) and the *tri*-phosphorylated structure (*m/z* = 1915; i.e. 1792+123) (Figure 4). The NMBΔ*lptA* mutant lacked all of these ions, consistent with the loss of LptA activity. To compare the PEA level in all of these strains, all of the lipid A molecules with or without PEA were included in the calculations. The ratio of total peak area of the lipid A molecules with PEA (i.e. *m/z* 1557+1653+1755+1807+1835+1857+1889+1897+1915 ions) to those without PEA (i.e. the total peak area of the *m/z* 1434+1530+1632+1684+1712+1734+1766+1774+1792 ions) was 2∶1 for wild-type strain NMB and 0.006∶1 for NMBΔ*lptA* in which almost all PEA was absent. In comparison, NMBΔ*dsbA1/dsbA2* had a ratio of 2∶1 similar to wild-type while NMBΔ*dsbA3* and NMBΔ*dsbA1/dsbA2/dsbA3* each had a reduced ratio of 1.7∶1. Therefore, it appears that DsbA3 contributes to the activity of LptA *in vivo*, which is consistent with the 3-fold decrease in polymyxin MIC observed for the DsbA3 mutants. Since there was no change in the ratio of PEA –bearing lipid A in the NMBΔ*dsbA1/dsbA2* relative to the parental isolate, this would suggest that the disulphide bonds catalysed by DsbA3 alone are sufficient to ensure full activity of LptA.

## Discussion

Neisserial LptA is an integral membrane protein which contains five disulphide bonds and has a predicted molecular weight of approximately 60 kDa. In *E. coli*, the stability and activity of neisserial LptA::His_x6_ was entirely dependent upon the co-expression of the EcDsbA oxidoreductase. Although expression of LptA::His_x6_ and EcDsbA in *E. coli* resulted in a 32-fold increase in polymyxin resistance, the proportion of lipid A headgroups substituted with PEA rose to 26%. It is unclear why expression of LptA did not result in 100% substitution of lipid A headgroups, but this may reflect the ability of neisserial LptA to recognise *E. coli* lipid A as a substrate. MALDI-TOF MS analysis of the *E. coli* lipid A confirmed that LptA modified lipid A headgroups with PEA, albeit only on one site. Due to the low prevalence of the ion substituted with PEA in these samples, an unambiguous allocation to the 1 or 4′ position could not be made by NMR (data not shown). However, in meningococci, lipid A is substituted at both the 1 and 4′ positions with PEA and both substitutions are lost in the absence of LptA [17]. Therefore, the attachment of PEA to one site of *E. coli* lipid A may be the result of specificity for the acyl-chain distribution on the lipid A. While the meningococcal lipid A possesses a lauroyl residue at the 2′ position of each glucosamine [41], *E. coli* LPS contains a lauroyl and myristoyl residue at the 2′ and 3′ positions of the distal glucosamine residue respectively, resulting in an asymmetrical acylation pattern of lipid A [42]. Therefore, it is possible that the proximal glucosamine of *E. coli* lipid A which resembles the lipid A of meningococci could act as an acceptor resulting in substitution of the 4′ position only. Alternatively, previous studies by Reynolds et al. [43] using EtpB in *E. coli* have demonstrated that acyl chain length and saturation of phosphatidylethanolamine determine which isoform is used as a substrate for the transfer reaction of PEA to lipid A. *E. coli* EtpB preferred phosphatydylethanolamine with C16 and C18 acyl chains, while phosphatydylethanolamine with C12 and C14 chains were not used by this transferase. Not surprisingly, in *E. coli* phosphatydylethanolamine isoforms with C12 and C14 acyl chains represents less than 5% of phosphatydylethanolamine present in the membranes [43]. In comparison, neisseria have membranes enriched with phosphatydylethanolamine isoforms carrying C12:0, C14:1 and C14:0 acyl chains which correspond to the length of the acyl chains decorating lipid A [44]. Therefore, if neisserial LptA::His_x6_ has a substrate preference for phosphatydylethanolamine isoforms with C12 and C14 acyl chains, the availability of this substrate would be a limiting factor for LptA::His_x6_ activity in *E. coli.*

Unlike *E. coli*, *Neisseria* spp. possesses three oxidoreductases which are considered to be specialist catalysts with different, although largely uncharacterised substrate specificity. DsbA1 and DsbA2 have been demonstrated to have some overlapping substrate specificity and are known to be responsible for stabilising the PilQ secretin and the PilE shaft proteins of Type IV pili [26]. DsbA3 which shares 57% and 51% amino acid identity with DsbA1 and DsbA2, respectively, is unable to oxidise the DsbA1/2 substrates and possesses a variant catalytic motif of CVHC rather than the canonical CPHC motif in DsbA1 and DsbA2 [24]–[26], [45]. Regardless of their functional and structural differences, all three oxidoreductases have similar redox potentials of −80 mV [46]. To determine whether any of these oxidoreductases contributed to the polymyxin resistance phenotype, each locus was inactivated in meningococcal strain NMB and led to a 3-fold increase in sensitivity to polymyxin for each mutant. Combinatorial mutations suggest that the defect in polymyxin resistance was additive, indicating that either each oxidoreductase affected a different pathway contributing to polymyxin resistance or they were co-operatively involved in stabilising proteins in a common pathway necessary for resistance. Apart from modification of lipid A with PEA groups, polymyxin resistance in meningococci and gonococci is also determined by the activity of resistance/nodulation/division (RND)-type efflux pump [47], [48] and indirectly by mutations affecting membrane barrier function [13]. Future analysis of these pathways will determine if there are oxidoreductase-dependent substrates involved in these mechanisms of resistance to polymyxin.

Correlates were sought between polymyxin sensitivity, LptA stability or activity and the presence of each of the neisserial oxidoreductases. Due to the low levels of natural expression of LptA in meningococci, the amount of LptA in the oxidoreductase mutants could not be detected by western immunoblot (data not shown). Expression of LptA::His_x6_ in meningococci enabled the detection of the oxidation of the protein in the presence of natural levels of each oxidoreductase. LptA remained oxidised in the presence of DsbA1, DsbA2 or DsbA3 alone. The presence of PEA on the lipid A headgroups was marginally reduced in the absence of DsbA3 but remained unaffected in the absence of DsbA1 and DsbA2 together. This implies that DsbA3 contributed to the activity of LptA, possibly through the donation of disulphide bonds to LptA. The disulphide bonds in the globular domain of LptA were found to create five small loops across the surface of the protein and are not involved in linking any of the core structures together that surround the active site [22]. Therefore, it is more likely that these disulphide bonds may be involved in protecting LptA from proteolytic degradation. It appears from this study that multiple oxidoreductases can donate disulphide bonds to LptA but only those donated by DsbA3 have some effect on LptA activity suggesting that each oxidoreductase may be responsible for different bonds.

Although the effect of disulphide bonds on LptA stability was accentuated in *E. coli*, the disulphide bonds did not appear to be essential for LptA stability in the neisserial periplasm. This is demonstrated by the observation that the triple Δ*dsbA1/dsbA2/dsbA3* knockout in *N. meningitidis* possessing none of these oxidoreductases is still 84-fold more resistant to polymyxin than a meningococcal strain in which LptA was inactivated. The analysis of the lipid A substitution profile of NMBΔ*dsbA1/dsbA2/dsbA3* confirmed that PEA addition to lipid A headgroups was present at approximately 63% of the wild-type levels and was similar to that of a DsbA3 mutant. This data suggests that even when LptA lacks the disulphide bonds donated by the oxidoreductases, the protein remains stable and mostly active. This was confirmed by the alkylation immunoblot of the NMBΔ*dsbA1/dsbA2/dsbA3* expressing LptA which revealed that reduced LptA was accumulated in the absence of all three oxidoreductases and was not completely degraded as it was in *E. coli*. Currently, it is not clear whether there is a difference in the relative abundance of proteases in the periplasm of each species. However, if the neisserial periplasm had reduced proteolytic activity relative to the periplasm of *E. coli*, the reduced LptA would appear to accumulate in the neisserial host background. Other factors which may affect protein stability include the ability to form part of a protein complex which could protect LptA from degradation in *N. meningitidis* but not *E. coli* as these partners would be absent in the heterologous host.

In conclusion, this study has shown that post-translational modification of LptA with disulphide bonds is an important mechanism governing stability and activity of the enzyme in the *E. coli* periplasm. Although neisserial LptA is also oxidised in the presence of all three specialised neisserial oxidoreductase catalysts, DsbA1, DsbA2 and DsbA3, the absence of these enzymes does not result in the complete loss of LptA stability and minimally reduces activity. This suggests that LptA is reasonably stable in the absence of any of these known oxidoreductases and may suggest other factors could be involved in determining the stability of LptA in the periplasm. Although the oxidoreductases were not essential for LptA activity or stability in *N. meningitidis*, they appear to contribute to other mechanisms that determine polymyxin resistance.

## Supporting Information

Figure S1 LptA::His_x6_ expression in whole cell lysates results in an intact protein of 60 kDa. Standardised cell lysates were separated by SDS-PAGE, followed by transfer to a membrane and western immunoblot using anti-His_x6_ HRP conjugate antibody to detect the presence of LptA::His_x6_. Lane 1 contains a whole cell lysate prepared from CKNM216 (strain NMB expressing LptA::His_x6_ from pCMK1001); Lane 2 contains 250 ng of LptA::His_x6_ purified from *E. coli* JCB571 expressing LptA::His_x6_ and *Ec*DsbA (CKEC564) and Lane 3 contains the New England Biolabs 2-212 protein standard. When compared to this standard, LptA::His_x6_ was detected as protein of approximately 60 kDa, consistent with the purified LptA::His_x6_ which has been confirmed to be full-length by solving the crystal structure (data not shown).
(TIF)

Figure S2 **LptA::His_x6_ is expressed from the shuttle vector pCMK1001 in** ***Neisseria meningitidis*** **.** Standardised cell lysates were separated by SDS-PAGE, followed by transfer to a membrane and the Western immunoblots were developed using anti-His_x6_ HRP conjugate antibody (Panel A) or rabbit anti-LptA antibody (Panel B) to detect the presence of LptA::His_x6_ in the extracts. Lanes were: Lane 1: NEB ColorPlus prestained protein molecular weight standard; Lane 2: *N. meningitidis* strain NMB; Lane 3: NMB expressing LptA::His_x6_ from pCMK1001 (CKNM216); Lane 4: NMBΔ*lptA::aadA*; Lane 5: *E. coli* JCB571 expressing LptA::His_x6_ and *Ec*DsbA; Lane 6: *E. coli* JCB571 expressing *Ec*DsbA, and Lane 7: LptA::His_x6_ purified from neisserial membranes prepared from CKNM216 using nickel columns. NOTE: ColorPlus prestained protein molecular weight standard migrates aberrantly such that the intact 60 kDa LptA::His_x6_ appears smaller (refer to Figure S1).
(TIF)
