# Correction: The Role of Oxidoreductases in Determining the Function of the Neisserial Lipid A Phosphoethanolamine Transferase Required for Resistance to Polymyxin

**DOI:** 10.1371/journal.pone.0110567

**Published:** 2014-10-07

**Authors:** The PLOS ONE Staff

## Notice of Republication

This article was republished on September 22^nd^, 2014, to correct an error where an incorrect Data Availability Statement was published with the paper. Please download the PDF again to view the corrected article.
