# Supplementary figures and images for: The Role of Oxidoreductases in Determining the Function of the Neisserial Lipid A Phosphoethanolamine Transferase Required for Resistance to Polymyxin
Source: PLoS One. 2014 Sep 12;9(9):e106513. doi: 10.1371/journal.pone.0106513 (PMC4162559; doi:10.1371/journal.pone.0106513)

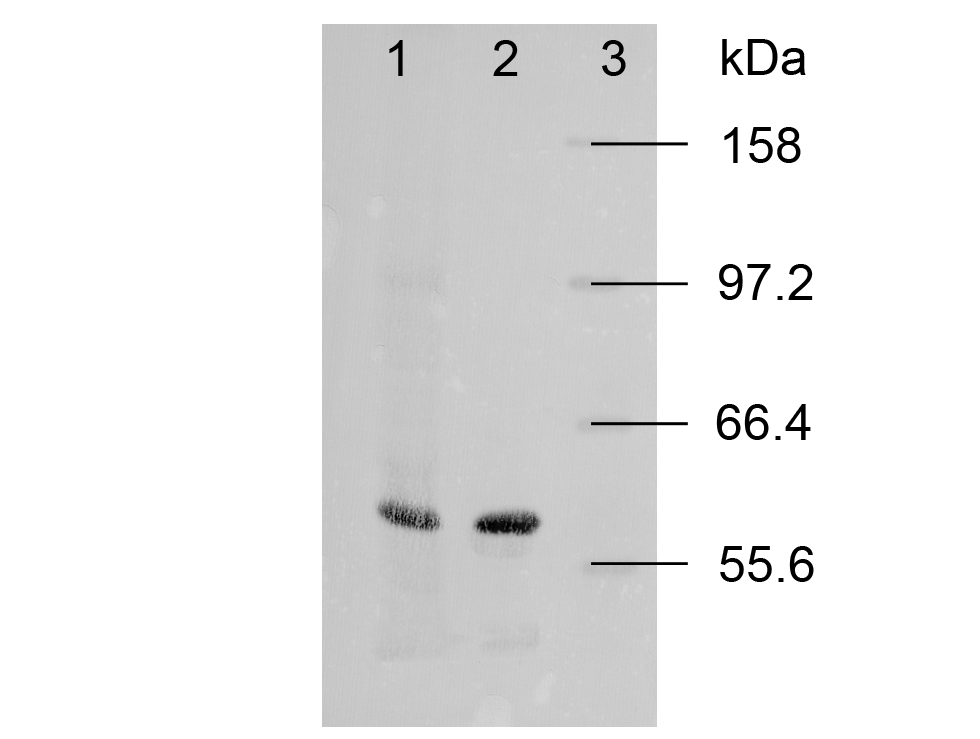

Supplement: Figure S1 — LptA::Hisx6 expression in whole cell lysates results in an intact protein of 60 kDa. Standardised cell lysates were separated by SDS-PAGE, followed by transfer to a membrane and western immunoblot using anti-Hisx6 HRP conjugate antibody to detect the presence of LptA::Hisx6. Lane 1 contains a whole cell lysate prepared from CKNM216 (strain NMB expressing LptA::Hisx6 from pCMK1001); Lane 2 contains 250 ng of LptA::Hisx6 purified from E. coli JCB571 expressing LptA::Hisx6 and EcDsbA (CKEC564) and Lane 3 contains the New England Biolabs 2-212 protein standard. When compared to this standard, LptA::Hisx6 was detected as protein of approximately 60 kDa, consistent with the purified LptA::Hisx6 which has been confirmed to be full-length by solving the crystal structure (data not shown). (TIF) [file pone.0106513.s001.tif]

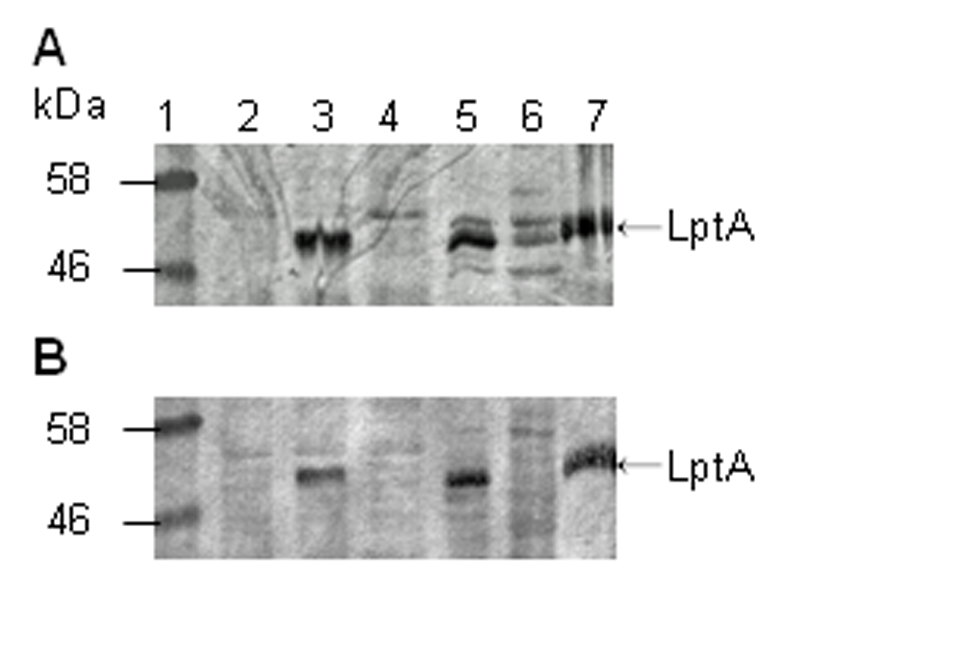

Supplement: Figure S2 — LptA::Hisx6 is expressed from the shuttle vector pCMK1001 in Neisseria meningitidis . Standardised cell lysates were separated by SDS-PAGE, followed by transfer to a membrane and the Western immunoblots were developed using anti-Hisx6 HRP conjugate antibody (Panel A) or rabbit anti-LptA antibody (Panel B) to detect the presence of LptA::Hisx6 in the extracts. Lanes were: Lane 1: NEB ColorPlus prestained protein molecular weight standard; Lane 2: N. meningitidis strain NMB; Lane 3: NMB expressing LptA::Hisx6 from pCMK1001 (CKNM216); Lane 4: NMBΔlptA::aadA; Lane 5: E. coli JCB571 expressing LptA::Hisx6 and EcDsbA; Lane 6: E. coli JCB571 expressing EcDsbA, and Lane 7: LptA::Hisx6 purified from neisserial membranes prepared from CKNM216 using nickel columns. NOTE: ColorPlus prestained protein molecular weight standard migrates aberrantly such that the intact 60 kDa LptA::Hisx6 appears smaller (refer to Figure S1). (TIF) [file pone.0106513.s002.tif]
